# Sex-Related Changes in the Clinical, Genetic, Electrophysiological, Connectivity, and Molecular Presentations of ASD: A Comparison between Human and Animal Models of ASD with Reference to Our Data

**DOI:** 10.3390/ijms24043287

**Published:** 2023-02-07

**Authors:** Asher Ornoy, Denis Gorobets, Liza Weinstein-Fudim, Maria Becker

**Affiliations:** 1 Adelson School of Medicine, Ariel University, Ariel 40700, Israel; 2 Hadassah Medical School, Hebrew University, Jerusalem 9112102, Israel

**Keywords:** ASD, human, rodents, clinical, pathophysiological, electrophysiological, genetic, molecular, sex differences

## Abstract

The etiology of autism spectrum disorder (ASD) is genetic, environmental, and epigenetic. In addition to sex differences in the prevalence of ASD, which is 3–4 times more common in males, there are also distinct clinical, molecular, electrophysiological, and pathophysiological differences between sexes. In human, males with ASD have more externalizing problems (i.e., attention-deficit hyperactivity disorder), more severe communication and social problems, as well as repetitive movements. Females with ASD generally exhibit fewer severe communication problems, less repetitive and stereotyped behavior, but more internalizing problems, such as depression and anxiety. Females need a higher load of genetic changes related to ASD compared to males. There are also sex differences in brain structure, connectivity, and electrophysiology. Genetic or non-genetic experimental animal models of ASD-like behavior, when studied for sex differences, showed some neurobehavioral and electrophysiological differences between male and female animals depending on the specific model. We previously carried out studies on behavioral and molecular differences between male and female mice treated with valproic acid, either prenatally or early postnatally, that exhibited ASD-like behavior and found distinct differences between the sexes, the female mice performing better on tests measuring social interaction and undergoing changes in the expression of more genes in the brain compared to males. Interestingly, co-administration of S-adenosylmethionine alleviated the ASD-like behavioral symptoms and the gene-expression changes to the same extent in both sexes. The mechanisms underlying the sex differences are not yet fully understood.

## 1. Introduction

Sex differences in the prevalences of many diseases are known phenomena. Such differences are more common in neuropsychiatric disorders, some of which have a significantly higher prevalence in males (e.g., ADHD and schizophrenia) and others in females (e.g., depression and bipolar disorder) [1]. These diseases generally have a genetic origin, with significant influence from the environment. Often, there are also differences in clinical presentations and/or in the effectiveness of treatments (e.g., depression) [2].

In the last few years, sex-related alterations in the clinical presentation of autism spectrum disorder (ASD) among preschool-age children have been described by many investigators [3,4]. However, some studies did not find sex-related differences [5] or observed behavioral alterations between boys and girls with ASD that were different from those generally described [6]. The sex differences in the clinical presentations of ASD also point to the possible need to use different tools in the diagnosis of ASD for boys and girls, or at least different diagnostic scores when using the same tools for boys and girls [7].

In rodents, genetic and non-genetic models of autistic-like behavior have been known for almost 40 years. However, generally, most studies were carried out on male mice and rats; thus, possible sex-related differences in ASD-like behavior were not ascertained. Neither were possible pathophysiological or genetic differences between sexes assessed. In the last few years, several studies have been published that have shown possible differences related to sex in animal models of ASD [8,9,10]. Differences were observed in the results of behavioral tests as well as in pathophysiological, electrophysiological, and gene-expression data.

The purpose of this review is to summarize the published data related to sex differences in all aspects of ASD in humans, as well as in rodent models of ASD-like behavior. In addition, we summarize our published studies on mice [8,9,10], in which ASD-like behavior was induced by prenatal and early postnatal valproic acid (VPA). In these and other studies, many findings differed between male and female animals, in ways surprisingly similar to those described in children with ASD.

## 2. Sex Differences in the Prevalence and Clinical Presentation of ASD in Children

Children with ASD exhibit communication and sociability problems and difficulties in interaction with others, have repetitive motor movements, seek specific sensory stimulation, and show excessive interest in a limited range of issues. They also often seek the accurate organization of items and have difficulties in adapting to changes [11].

Sex differences in the prevalence of ASD, which is 3–4 times higher in males, are well-established [5,12,13]. Sex differences also exist in several comorbid disorders, such as attention-deficit hyperactivity disorder (ADHD) and anxiety [14].

Several clinical symptoms seem to differ between male and female infants with ASD [5,15,16]. For example, communication problems are less common among girls with ASD compared to boys, while boys present with more restricted and repetitive behaviors and externalizing behavioral problems, including oppositional defiant disorder and greater impulsivity [17].

It is important to note that studies often differ in their results when investigating sex differences in the clinical presentations of children with ASD. This is apparently related to the diagnostic tools used, to the severity of ASD, and to the age at diagnosis [6].

There are many diagnostic tools for the assessment of neurobehavioral deviations in children and adults which are generally used in both sexes. An example is the Achenbach Child Behavior Checklist (CBCL). By using this questionnaire, it was observed that externalizing behavioral problems (e.g., impulsivity) are more common among boys and that internalizing problems (e.g., depression) are more common among girls with ASD [15,16]. Beggiato et al. [15] found, using the Autism Diagnostic Interview, Revised (ADI-R) questionnaire for the diagnosis of ASD, that the main differences between boys and girls with ASD are in the use of facial expressions for communication, which is better in boys, while girls engage better in imaginative play compared to boys with ASD. Unusual preoccupations and narrowly circumscribed interests are less evident in girls. The authors did not find lower intellectual abilities in girls. Mandy et al. [18] observed, in 325 children with high-functioning ASD, that there were no differences in verbal IQ but that females exhibited less repetitive movement and stereotyped behavior and that boys had more externalizing and social problems. However, there were similar social and communicative impairments. They also proposed that the lower intellectual abilities often observed in girls with ASD might not be evident in high-functioning ASD children.

Sex differences in clinical presentations point to the possibility that the methods used for the diagnosis of children with ASD may not be appropriate for both boys and girls and that they may be more appropriate for boys [18]. For example, if one uses the ADOS (Autism Diagnostic Observation Schedule), which is the method most commonly used to diagnose ASD, it may have to be adapted to females versus males. Indeed, girls are considered to have ASD with lower scores compared to boys [16]. This is also true for the CARS (Childhood Autism Rating Scale) diagnostic tool [19].

The few studies discussed above, as well as other studies, differ in their findings, making it difficult to delineate exactly all sex differences in the clinical presentation of ASD [20]. However, most studies agree that there are such differences and that the sex variations in the prevalence of ASD might also be related to these differences, making diagnosis in females more difficult. Moreover, these sex-related differences in symptoms lead to reduced prevalence of ASD in females, explaining, at least in part, their lower rate of ASD [21]. As the research on possible sex differences in the clinical presentations of ASD became more common, sex differences in other parameters that delineate ASD were outlined, as will be further discussed.

## 3. Sex Differences in Genetic Susceptibility to ASD

The heritability of ASD has been calculated as 85–92%, based on twin studies [22,23]. There seem to be distinct differences in genetic susceptibility between sexes. Females need a higher abnormal polygenic genetic load to show the symptoms compared to males [22]. For example, males with microdeletion of *SHANK1* have high-functioning ASD, while females with the same deletion are without ASD symptoms [22]. In male siblings of girls with ASD, the ASD is generally more severe than in male siblings of boys with ASD. However, *SHANK1*-deletion prevalence in males has been disputed, as Wang et al. [24] found one female with ASD symptoms with a de novo splice donor mutation involving the *SHANK1* gene. Numerous ASD-susceptible chromosomal loci and genes have been identified, suggesting a highly heterogeneous polygenic genetic architecture [25,26]. It is accepted that about 10–20% of ASD cases are related to defined rare mutations, genetic syndromes with highly penetrant chromosomal abnormalities, and de novo copy-number variants [26,27].

De novo mutations (DNMs) and risk genes for ASD have been identified among these cases and are considered important factors that contribute to the sex-related differences in terms of higher male versus female genetic liability, susceptibility, and development of ASD, in either sporadic or familial patterns [28,29]. The Simons Foundation Autism Research Initiative (SFARI) provides the most up-to-date database of candidate genes involved in autism susceptibility, the contents of which were integrated from multiple genetic studies and consist of more than 2000 DNMs and copy-number variant (CNV) loci (https://gene.sfari.org/autdb/CNVHome.do, accessed on 14 December 2022).

These DNMs correspond to the genes that are mostly associated with biological pathways related to chromatin remodeling, transcriptional regulation, and synaptic functions [30].

A female protective model was introduced to explain the liability for developing ASD and why a higher threshold of CNVs is required for females as compared with males [31,32]. Several studies reported a higher frequency of autosomal de novo CNVs in autistic females than in males with ASD (reviewed in [33,34]). Interestingly, in males, a higher frequency of rare deletions, such as microdeletions of approximately 555 kilobases on chromosome 16p11.2, was observed [35,36]. Thus, DNMs related to ASD etiology may also occur in non-symptomatic, normal individuals, especially females, who later transmit the mutations to male offspring.

The mammalian X chromosome differs from the autosomes due to its unequal representation in males (one copy) and females (two copies). Females have one inactivated copy of the X chromosome in every cell. Numerous disease conditions are associated with the X chromosome when recessive mutations on one of the maternal chromosomes are transmitted to males. Pinto et.al. [37] found that 4% of ASD-affected subjects had CNVs overlapping autosomal-dominant or X-linked genes and loci implicated in ASD and/or intellectual disability (ID) [37]. However, some genes (~10–15%) can escape X-inactivation [38].

Independent genome-wide studies on ASD susceptibility loci have demonstrated multiple ASD-susceptibility gene loci, including Xp22.3 and Xq13 on the X chromosome [32,39,40]. Rare mutations in two X-linked neuroligin genes, neuroligin 3 (*NLGN3*) and neuroligin 4X (*NLGN4X*), are linked to ASD and ID and contribute to the female protective model [41,42,43]. Neuroligins are cell adhesion molecules that are abundant in the postsynaptic membranes of glutamatergic synapses, playing an important role in the proper functioning and signal transmission of excitatory synapses [44]. Females with two X chromosomes will have two copies of *NLGN4X*, mutated and normal, whereas males have one copy of *NLGN4X* and one copy of *NLGN4Y*. Therefore, in males, even a single amino acid change in *NLGN4X* may lead to sex-linked autism.

Mutations in several genes and their association with ASD and distinct clinical phenotypes have been identified (reviewed in [26]). Deletion or point mutation of the X-linked gene encoding Methyl-CpG-binding protein 2 (*MECP2*) in females was observed in females with Rett syndrome [45]. Duplication of the *MECP2* gene in the Xq28 region has been found in both males and females with ASD [46,47]. The disorder predominantly affects males, but females who carry the duplication on one X chromosome may exhibit intellectual disability, sometimes similar to that seen in males. In addition, some genes may escape from X-inactivation [48,49]. Fragile X syndrome (FXS) is the leading monogenic cause of ASD [50], accounting for up to 3% of all cases, as about half of affected males show autistic behaviors [51]. Basu et al. [52] found a significant overlap with 28 fragile X messenger ribonucleoprotein 1 (FMR1) targets, including several well-studied autism candidate genes, such as *NLGN3, NRXN1, SHANK3, PTEN, TSC2*, and *NF1* [52].

## 4. Sex Differences in Brain Structure and Imaging Data in Humans with ASD

In recent years, more and more morphological and functional changes have been demonstrated in the brains of autistic children [53,54,55,56]. Several areas were shown to be affected in these children. More synapses, increased dendritic arborization, and abnormal concentrations of neurotransmitters, especially higher levels of dopamine, were demonstrated in the prefrontal cortexes of children with ASD. Some investigators have reported reduced brain volume and decreased cortical thickness in the frontal and temporal areas. A decrease in the number of Purkinje cells has been described in the cerebellum, and in the hippocampus, the site of continuous neuronal proliferation, increased cell apoptosis has been demonstrated. Abnormalities in functional connectivity in different areas of the brain have also been described. It is therefore not surprising that the brains of children with ASD or animal models of ASD-like behavior exhibit differences in electrophysiological characteristics when compared with brains of non-autistic individuals. It is also plausible that there could be sex differences in the morphology and electrophysiology of the brain in ASD. Indeed, a growing body of recent research has reported various sex differences between female and male brains at the cellular level, especially in brain circuitry and synaptic transmission, as will be discussed later (see Table 1).

Sex differences in brain images of children with ASD have been demonstrated by several investigators. For example, in twin studies, Cauvet et al. [55] found decreased cortical volumes and small surface areas in the temporal and frontal lobes in regions involved in social communication. These changes were more prominent in females with ASD as compared to males. The same pattern was observed in monozygotic as well as dizygotic twins. Nordalt et al. [56] found that ASD girls with internalizing problems, but not boys, have larger amygdala (see Table 1).

Several neuroimaging studies utilizing fMRI emphasizing the sex differences in brain connectivity in children with ASD have been published in the last few years [57,58]. In the first of the referenced studies [57], the investigators tried to reveal the correlations with social cognition (SoC) by Social Cognition–Multiple Choice (MASC) assessment in relation to changes in the social brain network. They studied 8 female and 10 male discordant twin couples. As a social brain network, the authors chose 20 regions of interest in the brain, bilaterally, including the insula, the middle frontal gyrus, and the lateral anterior fissure and estimated the cortical volumes, thicknesses, and areas of these regions. Initially, the authors compared the MASC scores of female and male individuals with ASD scores and found that there were no significant differences between them. More brain regions were involved in social cognition in males, but significant sex differences were observed only in the thicknesses of the angular and supramarginal gyri, which were larger in males, and only in dizygotic twin pairs [57]. The same group of investigators found in a twin study of 20 males and 12 females with ASD an association between brain neuroanatomy and restricted/repetitive movements. The severity and frequency of restricted movements were evaluated in terms of restricted and repetitive behaviors and interests (RRBIs). The authors focused on the thickness, volume, and surface area of 18 bilateral regions linked to the cortico-striatal loops; on the volume of the cerebellum; and on some subcortical regions, including the thalamus, the amygdala, and the putamen. Despite comparable differences in RRBIs between the twin pairs, the only neuroanatomical differences found were in females, who exhibited a significantly increased thickness of the right intraparietal sulcus. In males, increased volume of the globus pallidum was associated with RRBIs [58]. These findings demonstrate sex-related correlations between restricted movements and alterations in different brain networks: fronto-parietal networks in females and fronto-striatal networks in males. A subsequent study by Supekar and Menon [59], reporting on correlation of RRBIs and gray matter patterns of cortical regions in females and putamens in males, endorsed these results.

The sociability or social cognition paradigm is believed to be associated with the default mode network (DMN) which handles such categories as “me/myself” and “others” and has been implicated in autobiographical memories [63,64]. Various brain regions, such as the posterior cingulate cortex (PCC), the medial prefrontal cortex (mPFC), and the temporo-parietal junctions (TPJ), have been implicated in ASD. Lawrence et al. [54] analyzed resting-state fMRIs (rs-fMRIs) of 13-year-old autistic and typically developing girls and boys (nearly 50 individuals in each cohort). They found that, within the DMN, connectivity was not altered in the autistic children, nor in the typically developing children. The authors also investigated inter-network connectivity, including the bilateral fronto-insular cortex and the anterior cingulate cortex, together with the central executive network (CEN). In typically developing children, sex differences were observed only in the salience network, which is involved in decision making and behavioral coordination. In autistic children, there was substantially stronger connectivity in females as compared to males between the DMN and the CEN, and autistic females surpassed typically developing females in this parameter, whereas autistic males showed hyper-connectivity in the CEN and negative under-connectivity compared to healthy males [54].

Among the studies performed on the connectivity of networks linked to motor functions and thus with potential impacts on repetitive/restricted behaviors (RRBs), Smith et al. [60] compared cerebellar functional connectivity using rs-fMRI in autistic and typically developed (TD) 21-year-old males and females. At first, they applied “global connectedness” analysis to their data, demonstrating interactions between brain regions according to diagnosis-by-sex criteria. Both cerebellar clusters revealed decreased functional connectivity in ASD males compared to typically developing males, but increased patterns of connectivity in females.

It can therefore be concluded that autistic females are characterized by brain over-connectivity while males are characterized by under-connectivity [54,60,65,66]. However, the differences in the ages of the patients studied and differences in the study designs indicate the need for caution in drawing definite conclusions regarding sex differences in brain connectivity in ASD. We should also take into consideration that the sex differences are not static, as biological sex can impact the ASD phenotype differently during the whole lifespan, as demonstrated by several recent studies [67,68].

## 5. Sex-Related EEG Changes in Children with ASD (Table 1)

Electrophysiological changes associated with whole-brain activity are generally related to data collected by electroencephalography (EEG) in human patients. The classic evaluation of the functional differences revealed by EEG data is rooted in the separation of decomposed fast Fourier transform signals into several frequency bands: alpha, beta, gamma, delta, and theta [69,70,71]. Differences in these signals have been reported in a variety of neuropsychiatric disorders, such as ADHD, fragile X, Parkinson’s disease, and cognitive and memory function and dysfunction [61,72,73,74].

Abnormal oscillatory activity was found in various syndromes, ages, sexes, and experimental conditions. A comprehensive review of EEG power evaluation by Wang in 2013 summarized the main differences in the resting-state EEGs of ASD patients [71]. The authors pointed out several problems in the evaluation of the studies and explained the huge heterogeneity of these studies in terms of differences in patient sample size, presence or absence of intellectual disabilities, and age. However, some changes consistently prevail in the resting EEGs of ASD subjects. At first, the power spectra of EEGs in ASD are shifted [71]. Abnormal excessive powers are observed in delta, theta, beta, and gamma bands in the frontal, parietal, and right temporal areas of the brain [75,76,77,78,79], as well as in the occipital region in the case of gamma oscillations [80]. Alpha spectral power was found to be decreased in various brain regions of ASD patients [75,76,81], and the authors concluded that the spectral powers of the ASD patients as compared with the typically developing controls were U-shaped [71].

Recent findings conducted over the past 5 years corroborate the results accumulated earlier. For example, Paula et al. compared the power spectra of autistic children with those of healthy children (eight individuals in each group; one out of eight was female) during recognition of faces showing neutral, happy, and angry emotions. They found that the absolute powers of beta and gamma oscillatory bands were upregulated in the frontal, central, parietal, and occipital regions. Additionally, in frontal regions they observed an increase in the spectral power of the delta and theta bands in response to angry faces [82]. Likewise, Dickinson et al. evaluated peak alpha frequency (PAF)—a parameter reflecting the frequency at which alpha oscillations have the highest power, which is closely associated with developmental or cognitive functions in ASD—in 59 autistic versus 38 typically developing children exposed to visual stimuli in a dark room [83]. Peak alpha frequencies in ASD individuals were lower than in typically developing children in frontal, central, and occipital regions [83].

In a recent study by Gabard-Durnam et al. [84], the researchers tried to develop potential biomarkers for distinguishing ASD pathologies at an early age by tracking frontal-lobe EEG longitudinal changes during the first three years of age. Resting-state EEGs were acquired for infants aged 3, 6, 9, 12, 24, and 36 months with a high risk of ASD and having one or more older siblings diagnosed with ASD, and from low-risk infants (no older siblings diagnosed with ASD). All were without comorbid genetic syndromes, such as fragile X or tuberous sclerosis. Finally, by the age of 36 months, children were separated into three cohorts: high-risk familiar, ASD-diagnosed; familiar low-risk non-ASD; and high-risk with non-spectrum abnormalities (ADHD, anxiety, etc.). Analyzing their early-age EEG recordings, the authors referred to diminished gamma-band power at the age of 6 months as well as a lower rate of gamma power increase over the first three years as unique biomarkers for later ASD prediction. Moreover, within the same timeframe, summed delta powers showed steeper rates of increase in infants with ASD, distinguishing them from high-risk familiar ASD children later diagnosed without ASD. Notably, the authors mentioned that the sex of the participants was not a significant predictor of ASD in early-childhood EEG recordings [84].

There seem to have been only a few studies on sex differences using EEGs of children with ASD. Neuhaus et al. recorded resting-state EEGs of 142 children with ASD and compared them with those of 138 typically developed 8–18-year-old children, with nearly 50% females in each group [85]. Consistent with previous findings, they observed lower alpha-band power in the ASD group. They then evaluated power in beta, theta, alpha, beta, and gamma bands in relation to sex and found lower powers in ASD females in all the oscillatory frames around the whole scalp. Male children with ASD, but not females, demonstrated lower alpha and theta powers in the context of enhanced social abilities. A higher gamma power in the resting state was linked in males to reduced non-verbal IQ and more repetitive/restricted movements [85]. The authors concluded that all three main symptomatic aspects—socialization, cognition, and repetitive movements—correlated differently with EEG power.

Another clinical study was conducted recently with patients with FXS [62]. Resting-state EEG recordings revealed that males with FXS exhibit low individual peak alpha frequencies (IAPFs) in comparison to typically developing males. Such differences were not found in fragile X females when compared to healthy females [62]. This finding was also corroborated by Wang et al. [86]. Similar findings of PAF differences in typically developing versus autistic males and females were also obtained by magnetoencephalography (MEG) [87]. In the study, the authors did not find any significant differences in ASD females versus males and decided to evaluate PAF in correlation with SCQ results only in the male sample. The data are summarized in Table 1.

## 6. Sex Differences in Genetic Mouse Models

Numerous models of knock-out/knock-in mice have been generated based on the various recently defined DNMs and potential risk genes for ASD in human patients [52,88,89,90]. In the SFARI gene database, the Mouse Models module provides integrated envelopment of the current findings at the molecular, cellular, and behavioral levels on ASD. The SFARI gene Animal Models module provides a list of more than 100 genetic mice with ASD phenotypes relevant to the clinical presentation of autism in humans.

The mouse genetic models may be classified into several categories according to the types of genetic defects: modeling of autism is associated with defined genetic syndromes due to mutations in a single gene, such as fragile X or Rett syndromes. Similarly, non-syndromic autism associated with pathological mutations in single genes, such as the *Neuroligin* or *SHANK* family genes, and CNVs associated with autism, such as 15q11-q13 or 16p11.2, have been described.

Generally, almost all studies on mouse models with known ASD risk genes and mutations have reported that mice with specific mutations or deletions demonstrated autistic-like behavior, from mild phenotypes to severe behavioral impairments [52,88,89,90]. However, only a few studies have reported ASD-like behavioral differences between males and females. Generally, the findings regarding sexual dimorphism in ASD-like behavior are related to male predominance as regards the severity of autistic features. These sex differences were found to be prominent in a number of behavioral paradigms that have been established to assess autism-like behaviors in mice, such as the three-chamber test and the ultrasonic vocalization test for social communication, self-grooming, marble burying, and hole-board nose poking to assess repetitive behaviors. In addition, differences in the batteries of tests used to assess the comorbid neuropsychiatric symptoms, such as anxiety behavioral tests and memory and learning tests for assessment of intellectual disability, have also been reported [90,91,92].

### 6.1. Sex Differences in Mice with Single-Gene Mutations: MECP2 and FMR1 Genes

#### 6.1.1. X-Linked Methyl CpG Binding Protein 2 (MECP2) Gene

Mutations in the *MECP2* gene, which is located at Xq28, and encodes the methyl CPG binding protein 2, were shown to be the genetic cause of Rett [45,93]. The *MECP2* gene is expressed in two isoforms of different lengths that are involved in gene transcription in neuronal cells [94]. *Mecp2* expression levels are sex- and time-dependent [95,96,97,98]. Thus, females were found to have higher expression levels than males in the amygdala and hypothalamus on postnatal day 1 (PND1) [97]. This difference in expression levels between the sexes disappeared by day 10 (PN10) [97]. In another study, this difference in protein expression in the rat prefrontal cortex (PFC) was observed from E14 to PN7 but disappeared at PN14 [98].

A transcriptome analysis comparing the cortex tissues and microglia of 22–24-week-old *Mecp2* knockout (KO) male and female mice revealed 149 differentially expressed genes (DEGs) for male mice and 430 DEGs for female mice [99]. The DEGs shared by both male and female mice were mainly related to transport processes. The main phenotypic dimorphism was related to body weight, with females tending to weigh less than males [100].

Many mouse models displaying the features found in patients with Rett syndrome have been generated. Among these models, the *Mecp2tm1.1—Bird* mouse line, which completely lacks the MECP2 protein product [101], and the *Mecp2tm1.1—Jae* line, which expresses small MECP2 protein fragments [102], are the ones that have been most frequently investigated. Hemizygous male *Mecp2*-null mice are phenotypically normal until 4 weeks of age when they develop a Rett-like phenotype consisting of hind-limb clasping, tremors, breathing irregularities, loss of muscle tone, and hypoactivity. In addition, these mice develop metabolic abnormalities, increased serum cholesterol and triglycerides, and increased oxidative stress (reviewed in [103]). They also display reduced brain weight and body weight, experience a rapid phenotypic regression, and die between 6 and 12 weeks of age. Female mice heterozygous for *Mecp2* deletions develop the same features at 4–6 months of age and typically live a normal lifespan.

#### 6.1.2. X-Linked Intellectual Disability FMR1 Gene (FMR1)

FXS represents the most common monogenic form of ASD associated with an unstable expansion of a CGG trinucleotide repeat within the 5′ untranslated region (5′UTR) of the *FMR1* gene. This results in the loss of the fragile X messenger ribonucleoprotein 1 (FMR1), an RNA-binding protein that regulates protein-synthesis-dependent synaptic plasticity [104]. FMR1 is present in the brain in proximal dendrites and axons of neuronal cell bodies and is mainly associated with polyribosomes [105,106,107].

Mutant *Fmr1* KO mice [108,109] and rats [110,111] display altered social interaction and social play behavior, social anxiety, defects in visual attention and auditory dysfunctions, cognitive deficits, repetitive behaviors, and hyperactivity mimicking fragile X syndrome in humans. Nolan et al. [112] found that *Fmr1* gene deletion produces sex-specific behavioral changes. Thus, *Fmr1* KO homozygous females displayed increased repetitive behaviors when tested in the nose-poke test and enhanced motor coordination on the accelerating rotarod compared to wild-type females, while *Fmr1* KO males showed only hyperactivity in the open field [112]. However, evaluation of autistic-like behaviors in heterozygous *Fmr1* KO female mice revealed abnormal sociability at infancy and at the juvenile stage [113,114]. Follow-up observation at adulthood revealed that these abnormal behaviors of *Fmr1* KO female mice disappeared, demonstrating the temporal pattern of autistic-like behavior in females [113].

### 6.2. Non-Syndromic Autism Associated with Pathological Mutations in Single Genes

Male-preponderant behavioral deficits have been reported in many studies, using various models related to pathological mutations in single genes [115,116,117]. Sex dimorphism was reported in mice bearing mutations in the *Chd8* gene, which encodes a chromatin remodeling factor and modulates gene expression. *Chd8*-mutant mice (knock-in) with a human C-terminal protein-truncating mutation (*N2373K*) demonstrated male-preponderant behavioral deficits, evaluated at birth, juvenile, and adult stages [115]. Moreover, these ASD-like behaviors were accompanied by elevated neuronal activation in the hippocampus and prefrontal cortex under stressful conditions and changes in pups in ultrasonic vocalization [115] as well as altered neuronal, synaptic, and transcriptomic phenotypes [117,118]. In contrast, reduced brain baseline activity was found in females, which normalized upon maternal separation [115]. In neuronal-subset-specific (*NS-Pten*) knockout male and female pups, the analysis of ultrasonic vocalizations measured on postnatal days 8 and 11 revealed sex dimorphism in call types, vocalization duration, amplitude, and fundamental frequency, but both sexes had equal numbers of emitted calls [119].

Male predominance was also reported in studies using the *Btbr T+ tf/J* inbred mouse model of ASD, which mimics the core symptoms of ASD [120]. Significantly severe ASD-like behaviors, such as sociability and social communication, ultrasonic vocalizations, marble burying, and self-grooming, were observed in male *BTBR* mice more frequently than in female mice [121,122]. Sex dimorphism was also reported in studies with heterozygous and homozygous female and male mice [123] harboring a C-terminal deletion of Shank 3 at exon 21 (*Shank3tm1.1Pfw/J*), a mutation that is also found in humans [123,124].

Investigation of telomerase reverse transcriptase (*Tert*) overexpression in mice also demonstrated sex-dependent autistic-like effects. *Tert*-tg male mice demonstrated decreased sociability and social novelty preferences, reduced anxiety behavior, and decreased electro-seizure thresholds compared to females. In the prefrontal cortexes of *Tert*-tg male mice, increased expressions of postsynaptic NMDA and AMPA receptor subunit proteins were observed, though apparently not in females [125]. The data are summarized in Table 2.

## 7. Sex-Related Electrophysiological Changes in Rodents with ASD-like Behavior

There have been only a few electro-physiological studies on animal models of ASD demonstrating sex differences at the cellular or single-circuit levels. Recent research performed by Bódi et al. using a prenatal valproic acid (VPA) model of autism found some dissimilarities in hippocampal synaptic transmission between males and females [126]. Pregnant rats were administered intra-peritoneally a single dose of 500 mg/kg VPA on gestational day 12.5. Acute hippocampal slices were prepared in the offspring at 6 weeks and 3 months of age. In whole-cell patch clamp recordings in CA1 pyramidal cells of the hippocampus, they found that in 6-week-old males and females there were no differences in rheobase (i.e., a minimal current, which required depolarization of the cell) and relative cumulative spike number. However, in 3-month-old VPA-treated males, but not females, they observed a higher cumulative spike number and lower rheobase, demonstrating their higher excitability. Then, they evaluated the amplitudes, areas, and frequencies of spontaneous excitatory postsynaptic currents (sEPSCs) to assess spontaneous release. VPA treatment in female offspring did not alter the areas or amplitudes in either age group. However, the frequencies of sEPSCs in 3-month-old females were lower than in the controls. In 6-week-old male offspring they observed a decrease in sEPSC amplitudes, and the thresholds of population spikes and field excitatory postsynaptic potentials (fEPSPs) were significantly lower than in the controls, demonstrating the higher excitability of such neurons. Finally, they studied the early phase of long-term potentiation (eLTP) using theta-burst stimulation (TBS) and found a slight tendency toward eLTP decrease in 3-month-old VPA-exposed females [126].

Mice with *Tert* overexpression demonstrated ASD-like behavior [127]. In this model, the behavioral abnormalities, such as social impairment, nest-building impairment, and impaired LTP and spatial learning, are more specific for male mice than for females. In addition, overexpression of vesicular glutamate transporter 1 (vGLUT1) was observed in both males and females, with a slight increase in males [125]. Electrophysiological findings demonstrated enhanced glutamatergic transmission in mice with Tert overexpression, as male offspring had higher amplitudes of mEPSCs and AMPA/NMDA ratios [128].

## 8. Sex-Related Differences in Non-Genetic Animal Models of ASD: Valproic Acid-Induced Autistic-like Behavior in Rodents

Among the non-genetic models of ASD-like behavior in rodents, that produced with prenatal valproic acid (VPA) administration is the one most commonly used. VPA is a known human teratogen and is also teratogenic in rodents, inducing neural tube defects (NTDs), in addition to other malformations. It is important to administer the VPA to the pregnant rodents post active organogenesis, avoiding the possibility that VPA will induce embryonic malformations. Hence, VPA is usually given in the prenatal model not earlier than day 12 of gestation [129,130].

Only a few studies have tried to assess possible sex differences in the presentation of the ASD-like symptoms in these models. Even fewer studies have looked at possible differences in gene expression or brain structure/function between male and female rodents with ASD-like behavior. Some researchers have used only males, to eliminate possible sex differences [131,132,133,134,135,136,137,138] in behaviors, such as anxiety-related behaviors and repetitive behaviors [139,140]. Kataoka et al. [140] found that prenatal VPA in mice induced transient histone hyper-acetylation in the brains of offspring of male and female mice. However, social impairment at 8 weeks of age was observed only in male offspring.

Kazlauskas et al. [141] treated pregnant mice with 600 mg/Kg VPA on day 12.5 of pregnancy and tested in male and female offspring sociability as well as other neurobehavioral tests typical for ASD-like behavior. They found impaired sociability only in males, but the results of several behavioral tests were similar for both sexes. Moreover, the neuroinflammatory markers studied in the cerebellum and dentate gyrus were abnormal in both sexes but more severe in females.

Very recently, Ghahremani et al. [142] reported that female rats prenatally exposed to VPA performed better than VPA-exposed male offspring in Morris water-maze acquisition, which was conducted for spatial learning and memory analysis. Interval motor training (IT) and continuous training (CT) improved the cognitive functioning of VPA-exposed offspring of both sexes, with better results in females.

Morphological changes, such as a decrease in the number of Nissl-positive cells, have been reported in the somatosensory cortex of VPA-exposed male but not female offspring and may be involved in the sex-dependent social interaction deficits in mice [143].

Knopko et al. [144] found that VPA administration to pregnant dams on gestational day 12.5 induced robust and widespread histone lysine acetylation at specific exons of the Bdnf gene in the brains of offspring. They also reported differences in mRNA levels of Bdnf transcripts between males and females, which may be involved in the female protective effect in ASD. Kim et al. [98] reported a male-specific decrease in MeCP2 in the prefrontal cortex, MeCP2 being involved in transcriptional regulation via histone acetylation and chromatin structure. In addition, MeCP2 knockdown resulted in an increase in PSD95, an important postsynaptic protein, in neural progenitor cells derived from males but not females. Gu et al. [145] reported remarkable alterations in the gut microbiota and fecal metabolites of VPA-exposed rat offspring with sex-specific differences. They proposed that future treatment of ASD may differ between the sexes. Scheggi et al. [146] treated prenatally VPA-exposed rats with a fenofibrate (a peroxisome proliferator-activated receptor α agonist)-enriched diet and found effective relief of social impairment and perseverative behavior only in females.

## 9. Behavioral and Molecular Studies on Early Postnatal and Prenatal VPA Administration in Mice. (These Studies were Published by us Previously [8,9,10])

VPA is a known epigenetic modulator, and this characteristic might be responsible for the VPA-induced ASD-like behavior. We therefore performed neurobehavioral and molecular studies in ICR mice treated either prenatally or early postnatally with VPA [129,130,147]. We hypothesized that by using an antagonistic epigenetic modulator—S adenosyl-methionine (SAMe)—we might reverse many of the ASD-like behavioral changes induced by VPA. We also hypothesized that many of the clinical and/or pathophysiological features induced by VPA, including expected changes in gene expression, would differ between male and female mice. On the basis of behavioral studies that differed between male and female mice, we also thought that there might be changes in gene expression, with differences between sexes.

### 9.1. Postnatal Administration of VPA—Behavioral Studies

In our postnatal studies [8,9], we treated 4-day-old mouse offspring with 300 mg/Kg body weight VPA by intraperitoneal injection and studied the ASD-like behavioral and molecular changes. In order to examine whether alleviation of the ASD-like symptoms is also sex-dependent, we added, on postnatal days 5, 6, and 7, 30 mg/Kg SAMe by intragastric lavage or normal saline.

We first recorded weight, growth, and neurobehavioral development. During postnatal days 50–59, we carried out a variety of behavioral tests used for the assessment of ASD-like behavior, and on day 60 we sacrificed the animals and performed our biochemical and molecular studies on the prefrontal cortexes. We compared all studied parameters between males and females [8].

VPA induced in males and females higher grooming frequency, which also means reduced sociability. However, sociability was less impaired in females compared to males. Higher anxiety was observed in VPA-treated females compared to treated males. Increased anxiety (less head dipping) in females and males treated with VPA was also observed in the elevated plus-maze test. VPA-induced anxiety was significantly higher in females compared to males. Analysis of memory from the water T-maze test showed that VPA reduced memory only in females. The results of the three-chamber test for sociability testing showed reduced sociability in males but not in females. All these behavioral differences between male and female mice were very similar to the behavioral differences reported between girls and boys with ASD. All the above-described behavioral changes were alleviated in both sexes by the concomitant administration of SAMe.

Increased brain oxidative stress was observed in the brains of children with ASD as well as in animals with ASD-like behavior [7,148]. We found that VPA-treated females showed decreased activity of the antioxidant enzyme superoxide dismutase (SOD), increased activity of Catalase (CAT), and increased Malone-dialdehyde (MDA) as signs of increased oxidative stress. This was corrected by co-administration of SAMe. In males, VPA only increased the activity of SOD, implying that oxidative stress was less prominent compared to females. The data are summarized in Table 3.

#### Studies of Gene Expression in the Brain

We used the nCounter neuropathology panel which measures the expression of 770 genes in the brain. These are neural inflammatory genes, neurogenesis genes, and genes involved in various biological pathways, such as the vascular endothelial growth factor (VEGF) pathway, among others. VPA induced a variety of changes in gene expression in the brains of both sexes. There were sex differences in the VPA-induced changes in gene expression. In females, more genes were changed by VPA, and half of the genes were upregulated and half of them were downregulated. In males, most genes the expression of which was changed by VPA were upregulated [9]. SAMe reversed the changes in 75% of the genes in males and 52% in females. Changes of over 50% were observed in the expression of neuroinflammation, neurogenesis, and general biological genes. Most of these changes were reversed by oral SAMe. SAMe alone did not change gene expression [9].

### 9.2. Prenatal Studies

We treated ICR mice on day 12 of gestation with a single dose of 600 mg/Kg of VPA and administered SAMe on days 13–15, at a dose of 30 mg/Kg/day. Treated mice were allowed to deliver, and the offspring were followed up to two months of age.

Behavioral studies were performed during postnatal days 55–60, using methods similar to those used in the early postnatal animals. We found that VPA induced ASD-like behavioral changes similar to those observed in the animals treated during early postnatal life. VPA reduced sociability, increased anxiety in both sexes, and reduced learning abilities, with differences between males and females. SAMe only partially alleviated the ASD-like behavioral changes caused by prenatal VPA, thus being less effective than in the early postnatal treatment model.

We studied gene expression in the anterior parts of the cerebral hemispheres (mainly the prefrontal cortexes) only in day-one offspring, using the Nanostring nCounter neuropathology panel [10].

VPA did not induce any changes in gene expression of the brains of male or female neonates. In contrast, SAMe produced changes in the expression of many genes in the cerebral hemisphere. There were sex differences in the genes with changed expression levels, as more genes were changed in females compared to males. Interestingly, concomitant prenatal administration of VPA prevented most of the gene-expression changes induced by prenatal SAMe.

## 10. Possible Explanation of the Sex Differences in ASD in Humans

Since the major difference between males and females is the difference in sex chromosomes, along with numerous sex-linked genes, many studies that have tried to explain the differences in the prevalence and severity of various diseases between males and females have looked at X and Y chromosomes.

An important question, therefore, is whether ASD is a sex-linked disorder, with the X chromosome reducing its prevalence. Most studies have demonstrated that it is not, but there is a higher prevalence of ASD in children who have a lower proportion of X chromosomes. For example, in Turner syndrome, with a female phenotype and only one X chromosome, 3% of individuals have ASD—about three times the risk of the general population. In children with Klienfelter syndrome (XXY), 10% have ASD, and in those with XYY chromosomes about 20% have ASD. There is no increased risk in triple X (XXX) individuals. Hence, the Y chromosome is apparently a risk factor for ASD, or, alternatively, the X chromosome is a protecting factor. However, the authors give no explanation of why in Turner syndrome, with only one X chromosome but no Y chromosome, or in the case of XXY, with two X chromosomes, there is an increased risk for ASD.

Several investigators have attempted to explain the male predominance of ASD by sex-specific genetic changes in single-nucleotide polymorphisms (SNPs), single-nucleotide variants (SNVs), copy-number variants (CNVs), and different sex-biased proteins [22,23,149,150,151,152]. Mutations in specific genes, particularly genes linked to X or Y chromosomes, have been implicated in male vulnerability to ASD [44,153,154]. For example, Nguyen et al. [44] suggested that the X-linked cell adhesion molecule *NLGN4X,* a mutation in which is known to be associated with ASD or intellectual disabilities, may contribute to the male bias in ASD, because the Y chromosome variant, *NLGN4Y*, cannot compensate for the functional deficits created by the X-linked mutation. Others have hypothesized that male predominance in ASD occurs due to sex-specific steroidal or hormonal effects on pathways involving immune or inflammatory functions during critical periods of brain development [155,156,157]. Interestingly, in contrast to the male predominance reported in ASD prevalence, in children born to mothers treated with VPA during pregnancy there seems to be no sex difference in the rate of ASD [147].

An alternative theory for ASD sex-related changes suggests that ASD is an “extreme expression of the male brain”, as males have a higer tendency to systemize, while females have a higher drive to “empathize”. These behavioral differnces are the result of the effects of androgens (testosterone) on brain development in males, resulting in sex-related behavioral differences. Hence, the higher levels of testosterone during fetal development increase male vulnerability to ASD [158]. Evidence that intrauterine testosterone might play a role was obtained by measurements of amniotic fluid testosterone from amniocenteses. Amniotic fluid testosterone (of fetal origin) levels were inversely associated with eye contact in children at one year of age and inversely correlated with vocabulary development at two years and with the quality of social relationship and empathy at 4 years. In addition, testosterone levels were positively associated with narrow interests at 4 years and with a tendency to systemize at 8 years [150]. These are behavioral features that are sex-related and which often appear in ASD.

## 11. Conclusions

The prevalence of ASD, similar to several other neuropsychiatric disorders, is significantly higher in males compared to females. Moreover, there are also differences in clinical presentation and comorbidities between males and females. In the last few years, sex differences have also been observed in the electrophysiology, imaging, and connectivity of the brain. Sex-related differences are also reported in genetic and non-genetic models of autistic-like behavior in rodents. Some of these differences (i.e., behavioral and electrophysiological) are similar to those observed in humans. Moreover, such differences are also observed in autistic-like behavior induced by mutations in sex chromosomes in human or in animal models, implying that the differences are related to the different sex chromosomes. Although the exact mechanisms responsible for these differences are as yet unknown, it seems that they are related to hormonal or genetic changes that define the phenotype of the male or female. Additional studies are clearly needed to further understand the possible interaction between the various genetic entities (X or Y chromosomes and their genes) in the etiology of many diseases that are sex-related. Finally, due to the significant differences in the clinical presentation of ASD between girls and boys, there seems to be a need for the adjustment of diagnostic tests to detect or exclude ASD. There is a greater need for this in autism than in most other neuropsychiatric diseases, where the same methods of diagnosis are used for both sexes.

## Figures and Tables

**Table 1:** Sex differences in brain structures and functions evaluated by fMRI and EEG, respectively.

Author	Method of Study/Brain Region of Interest/Participants of Study	Sex Differences
Cauvet et al. [57]	fMRI in correlation with social cognition tasks. Various brain regions. Twin couples of adolescent age.	Angular and supramarginal gyri in autistic females showed smaller thicknesses than the corresponding regions in males.
van’t Westeinde et al. [58]	fMRI in correlation with repetitive or restricted behaviors (RBBs) movement evaluation. Various brain regions. Twin couples of adolescent age.	Autistic females showed significantly increased thickness of right intraparietal sulcus in association with RBBs. Autistic males exhibited a correlation between RBBs and increased volume of the globus pallidum.
Supekar and Menon [59]	Structural MRI with gray matter volume evaluation in association with RBBs and social functions in various brain regions. Typically developed and autistic girls and boys 10 years of age were chosen from the ABIDE dataset.	Autistic girls exhibired fewer RBBs than boys. Organization of gray matter was different for autistic males and females in the systems linked with motor regulation: motor cortex, crus I of cerebellum, and supplementary motor area. Regions linked with “social brain” angular and fusiform gyri, amygdala, and insula were also different. Gray matter organization in the right putamen was associated with severity of RBBs in males. Social brain differences were not associated with severity of symptoms of sociability.
Lawrence et al. [54]	fMRI with functional connectivity analysis of salience, central executive, and default mode networks. Nearly 50 autistic and typically developed children studied.	Overconnectivity in autistic females between DMN and CEN, whereas in males a decrease was observed.
Smith et al. [60]	Resting-state fMRI in correlation with RBBs, with analysis of intracerebellar connectivity. Twenty-one-year-old neurotypical and autistic males and females.	In autistic males: decreased pattern of connectivity; in females: increased connectivity. Both groups were compared with typically developed individuals.
Neuhaus et al. [61]	Resting-state EEG. Whole-scalp recording. Autistic and typically developed children 8–18 years old.	Autistic females’ oscillatory frames showed diminished power in comparison with those of males. Males possessed lower alpha and theta powers in correlation with enhanced sociability. Higher gamma power was associated with RBBs in males.
Smith et al. [62]	Resting-state EEG. Fragile X and typically developed children.	Fragile X male patients had lower peak alpha frequencies than typically developed children, without any differences in females.

**Table 2:** Sex differences in genetic mouse models.

Author	Specific Gene	Sex Differences
Jeon SJ et al. [99] Kerr, B. et al. [100]	*Mecp2* gene	The transcriptome analysis of cortex tissues and microglia of 22–24-week-old *Mecp2*-knockout (KO) mice revealed 149 differentially expressed genes (DEGs) for male mice and 430 DEGs for female mice. The DEGs shared by both male and female mice were mainly related to transport processes [99]. The main phenotypic dimorphism was related to body weight: females weighed less than males [100].
Nolan et al. [112] Gauducheau, M [113] Reynolds, C.D [114]	*Fmr1* gene	*Fmr1* KO homozygous females displayed increased repetitive behaviors when tested in the nose-poke test and enhanced motor coordination on the accelerating rotarod. *Fmr1* KO males showed only hyperactivity in the open field [112]. Heterozygous *Fmr1* KO female mice exhibited abnormal sociability at infancy and at the juvenile stage [113,114]. The abnormal behavior of *Fmr1* KO female mice has a temporal pattern of autistic-like behavior [113].
Andreae, L.C. et al. [115] Lee, S.Y. et al. [118] Jung, H. et al. [117]	*Chd8* gene	*Chd8*-mutant mice (knock-in) demonstrated male-preponderant behavioral deficits, evaluated at birth, juvenile, and adult stages [115]. ASD-like behaviors are accompanied by elevated neuronal activation in the hippocampus and prefrontal cortex under stressful conditions and changes in pups in terms of ultrasonic vocalization [115]. In female mice, reduced brain baseline activity was found, which normalized upon maternal separation, though not in males [115]. Altered neuronal, synaptic, and transcriptomic phenotypes were more prominent in male than in female mice [117,118].
Binder, M.S. et al. [119]	*Pten* gene	In neuronal-subset-specific (*NS-Pten*) knockout male and female pups, the analysis of ultrasonic vocalizations measured on postnatal days 8 and 11 revealed sex dimorphism in vocalization duration, amplitude, and fundamental frequency. Mice of both sexes emitted equal numbers of calls [119].
McFarlane et al. 2 [120] Schwartzer et al. [121] Amodeo, D.A et al. [122]	*Btbr* gene	Male predominance was also reported in studies using the *Btbr T+ tf/J* inbred mouse model of ASD [120]. Impairments were observed in sociability and social communication, ultrasonic vocalizations, marble burying, and self-grooming behaviors, these being observed more frequently in male than in female mice [121,122].
Matas, E et al. [123]	*Shank 3* gene	Gait parameters demonstrated more severe disturbances in *Shank3* ΔC/ΔC male mice than in females and were accompanied by decrease in the levels of cerebellar mGluR5 protein only in male mice [123].
Kim, K.C. et al. [125]	*Tert* gene	*TERT*-tg male mice demonstrated decreased sociability and social novelty preferences, reduced anxiety behavior, and decreased electro-seizure thresholds compared to females. In the prefrontal cortexes of *TERT*-tg male mice, increased expressions of postsynaptic NMDA and AMPA receptor subunit proteins were observed only in male mice [125].

**Table 3:** Sex differences in VPA-induced ASD-like behavioral characteristics.

Author	VPA Administration	Sex Differences
Kataoka et al. [140]	Prenatal day 12.5, 500 mg/kg, mice	Social interaction deficits (decrease in sniffing behavior) at 8 weeks of age were observed in male but not in female mice.
Kazlauskas et al. [141]	Prenatal day 12.5, 600 mg/kg, mice	VPA-exposed males but not females exhibited reduced sociability levels and a lack of preference for the social stimulus over a novel object, as well as increased basal corticosterone levels in response to an inflammatory stimulus.
Ghahremani et al. [142]	Prenatal day 12.5, 500 mg/kg, rats	VPA-exposed female offspring showed better performance than VPA-exposed male offspring in Morris water-maze acquisition, which represents spatial learning and memory.
Hara et al. [143]	Prenatal day 12.5, 500 mg/kg, mice	VPA-exposed female but not male offspring exhibited decreased Nissl-positive cell numbers in the prefrontal cortex.
Knopko et al. [144]	Prenatal day 12.5, 400 mg/kg, mice	VPA-exposed male and female offspring exhibited differences in mRNA levels of Bdnf transcripts, which may be involved in the female protective effect in ASD.
Kim et al. [98]	Prenatal day 12 400 mg/kg, rats	VPA-exposed male but not female offspring showed reduced levels of methyl-CpG-binding protein 2 (MeCP2) in the prefrontal cortex.
Gu et al. [145]	Prenatal day 12.5, 600 mg/kg, rats	Differentially abundant fecal metabolites and alterations in the gut microbiota reported in both male and female VPA-exposed rats, with sex-specific differences.
Scheggi et al. [146]	Prenatal day 12.5, 500 mg/kg, rats	Treatment of VPA-exposed rats with a fenofibrate-enriched diet, a peroxisome-activated receptor α agonist, reduced social impairment, and persistent behavior in females but not males.
Ornoy et al. [8]	Postnatal day 4, 300 mg/Kg, mice	Behavioral studies showed reduced sociability in VPA-treated males but not in females, while VPA-treated females exhibited higher anxiety and reduced memory compared to VPA-treated males. Sex differences in oxidative stress markers have also been reported.
Weinstein-Fudim et al. [9]	Postnatal day 4, 300 mg/Kg, mice	Gene expression analysis of VPA-treated pups revealed a wide range of gene expression changes with significant sex differences. Most of these changes were corrected by SAMe treatment.
Weinstein-Fudim et al. [10]	SAMe and VPA on prenatal day 12.5, 600 mg/Kg VPA, mice	VPA prevented SAMe-induced changes in gene expression in the brain in a sex-related manner.

## Data Availability

Not applicable.

## Abbreviations

ADHD	Attention-deficit hyperactivity disorder
ADI-R	Autism Diagnostic Interview, Revised
ADOS	Autism Diagnostic Observation Schedule
AMPA	α-amino-3-hydroxy-5-methyl-4-isoxazolepropionic acid
ASD	Autism spectrum disorder
CAT	Catalase
CBCL	Achenbach Child Behavior Checklist
CEN	Central executive network
Chd8	Chromodomain Helicase DNA Binding Protein 8
CNV	Copy-number variants
CT	Continuous training
DEGs	Differentially expressed genes
DMN	Default mode network
DNMs	De novo mutations
EEG	Electroencephalography
eLTP	Early phase of long-term potentiation
fEPSP	Field excitatory postsynaptic potential
FMR1	Fragile X Messenger Ribonucleoprotein 1
fMRI	Functional magnetic resonance imaging
FMR1	Fragile X messenger ribonucleoprotein 1
FXS	Fragile X syndrome
IAPF	Individual alpha peak frequency
IQ	Intelligence quotient
IT	Interval motor training
KO	Knockout
LTP	Long-term potentiation
MASC	Social Cognition–Multiple Choice
MDA	Malone-dialdehyde
MECP2	Methyl-CpG-binding protein 2
MEG	Magnetoencephalography
mEPSCs	Miniature excitatory postsynaptic current
mPFC	Medial prefrontal cortex
NF1	Neurofibromatosis type 1
NLGN3	Neuroligin 3
NLGN4X	Neuroligin 4X
NMDA	N-methyl-D-aspartate
NRXN1	Neurexin 1
NS-Pten	Neuronal-subset specific
NTD	Neural tube defects
PAF	Peak alpha frequency
PCC	Posterior cingulate cortex
PN	Postnatal day
PTEN	Phosphatase and tensin homolog
RRBs	Repetitive/restricted behaviors
RRBIs	Restricted and repetitive behaviors and interests
rs-fMRI	Resting-state fMRI
SAMe	S adenosyl-methionine
SCQ	Social Communication Questionnaire
sEPSCs	Spontaneous excitatory postsynaptic currents
SFARI	Simons Foundation Autism Research Initiative
SHANK1	H3 And Multiple Ankyrin Repeat Domains 1
SNPs	Single-nucleotide polymorphisms
SoC	Social cognition
SOD	Superoxide dismutase
TBS	Theta-burst stimulation
TD	Typically developed
TERT	Telomerase reverse transcriptase
*Tert*-tg	Telomerase reverse transcriptase -transgenic
TPJ	Temporo-parietal junctions
vGLUT1	Vesicular glutamate transporter 1
VPA	Valproic Acid
SNVs	Single-nucleotide variants
5′UTR	5′ untranslated region
